# Ancient Schwannoma of the Thumb Pulp Presenting as a Cystic, Non-enhancing Lesion on MRI: A Diagnostic Pitfall

**DOI:** 10.7759/cureus.115254

**Published:** 2026-08-26

**Authors:** Julia K Russolillo, Graham Grisedale, Tami D DenOtter, Clay Jarrell, Ryan Trowbridge

**Affiliations:** 1 Dermatology, Creighton University School of Medicine, Omaha, USA; 2 Radiology, Creighton University School of Medicine, Omaha, USA; 3 Pathology, Creighton University School of Medicine, Omaha, USA

**Keywords:** ancient schwannoma, dermatology case report, digital tumor, epidermal inclusion cyst, magnetic resonance imaging

## Abstract

Schwannomas are benign peripheral nerve sheath tumors that rarely occur in the digits, with the ancient (degenerative) variant being even more uncommon. We describe the case of a 59-year-old male with a recurrent, painless thumb pulp mass. Preoperative magnetic resonance imaging (MRI) demonstrated a cystic-appearing lesion without significant postcontrast enhancement, interpreted as an epidermal inclusion cyst (EIC). Intraoperatively, the lesion was larger than expected and extended to the periosteum of the distal phalanx. Complete excision revealed a schwannoma with degenerative (“ancient”) changes, confirmed by diffuse S100 and SOX10 immunoreactivity. This case provides MRI characterization of an ancient schwannoma of the thumb, demonstrating an atypical cystic, non-enhancing appearance*.* In this case, the lesion demonstrated atypical imaging features, leading to diagnostic confusion with an EIC. This report highlights a potential diagnostic pitfall of imaging and the importance of histopathology for definitive diagnosis, while expanding the spectrum of reported radiologic appearances of digital ancient schwannomas.

## Introduction

Schwannomas are common, benign peripheral nerve sheath tumors arising from Schwann cells [1]. They are often located in soft tissue along major peripheral nerves of the head, neck, or extremities. While typically slow-growing and asymptomatic, they may occasionally present with pain and paresthesia. Histologically, schwannomas alternate between tightly packed, fascicular areas (Antoni A) and loose, reticular areas (Antoni B) [2]. Elongated, palisading nuclei called Verocay bodies are common within Antoni A regions [3].

Ancient schwannomas are a rare subtype characterized by degenerative changes. They have been reported across a wide age range and occur most commonly in the pelvis/lumbar, abdomen, and head and neck regions [4]. They are very rarely reported to involve the digits of the hand [5,6]. In general, ancient schwannomas have been reported to range from 1.5 to 11 cm in size across all anatomical sites, with gross features including hemorrhage, mucoid change, and cystic degeneration [4]. Histologically, ancient schwannomas exhibit similar Antoni A and B patterns but are distinguished by diffuse hypocellularity and degenerative changes, including calcification, cyst formation, and hyalinization [7,8]. On MRI, conventional schwannomas are typically T1 iso- to hypointense, T2 hyperintense, and enhancing, whereas degenerative changes in schwannomas can result in more variable appearances and complicate preoperative diagnosis [7]. To our knowledge, detailed MRI findings of ancient schwannomas involving the digits have not previously been reported.

## Case presentation

**Figure 1:**
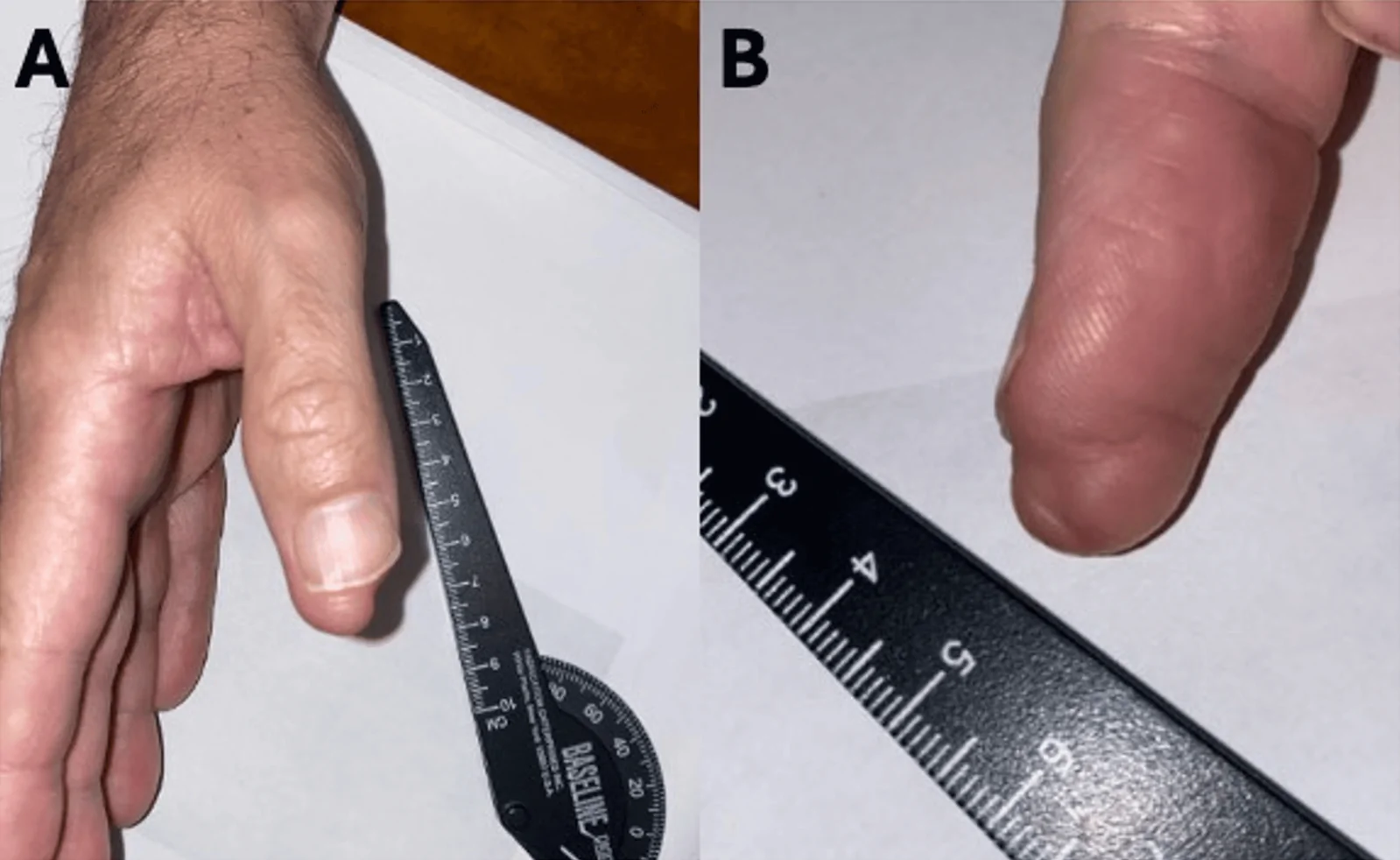
Clinical appearance of the thumb pulp mass. (A) Preoperative photograph demonstrating the location of the mass within the distal thumb pulp. (B) Close-up view of the mass with a ruler for scale.

A 59-year-old male presented with an approximately 1 cm, painless, rubbery mass on his right thumb (Figure 1). He denied any history of trauma. There was no personal or family history of schwannomatosis syndromes, including NF2-related schwannomatosis, and no additional lesions or associated symptoms suggestive of these conditions were identified [9]. The mass, although bothersome, did not interfere with activities of daily living.

The patient reported that the lesion had been drained approximately ten years earlier by another dermatologist but had recurred within a few months. Thereafter, the lesion remained relatively stable in size until presentation. Records from the previous procedure were unavailable, and it is unclear whether tissue was submitted for histopathologic examination. However, the patient recalled being told that there was no evidence of malignancy. The preoperative differential diagnosis included acral fibromyxoma, glomus tumor, or giant cell tumor of the tendon sheath. Given the lesion’s location and findings on palpation, the dermatologist considered epidermal inclusion cyst (EIC) as unlikely. Because of the lesion’s recurrence, bothersome presence, and size (1 cm) relative to the limited space of the thumb pulp, the patient elected to proceed with surgical excision. He was referred to an orthopedic hand surgeon and underwent preoperative imaging as part of the surgical evaluation.

**Figure 2:**
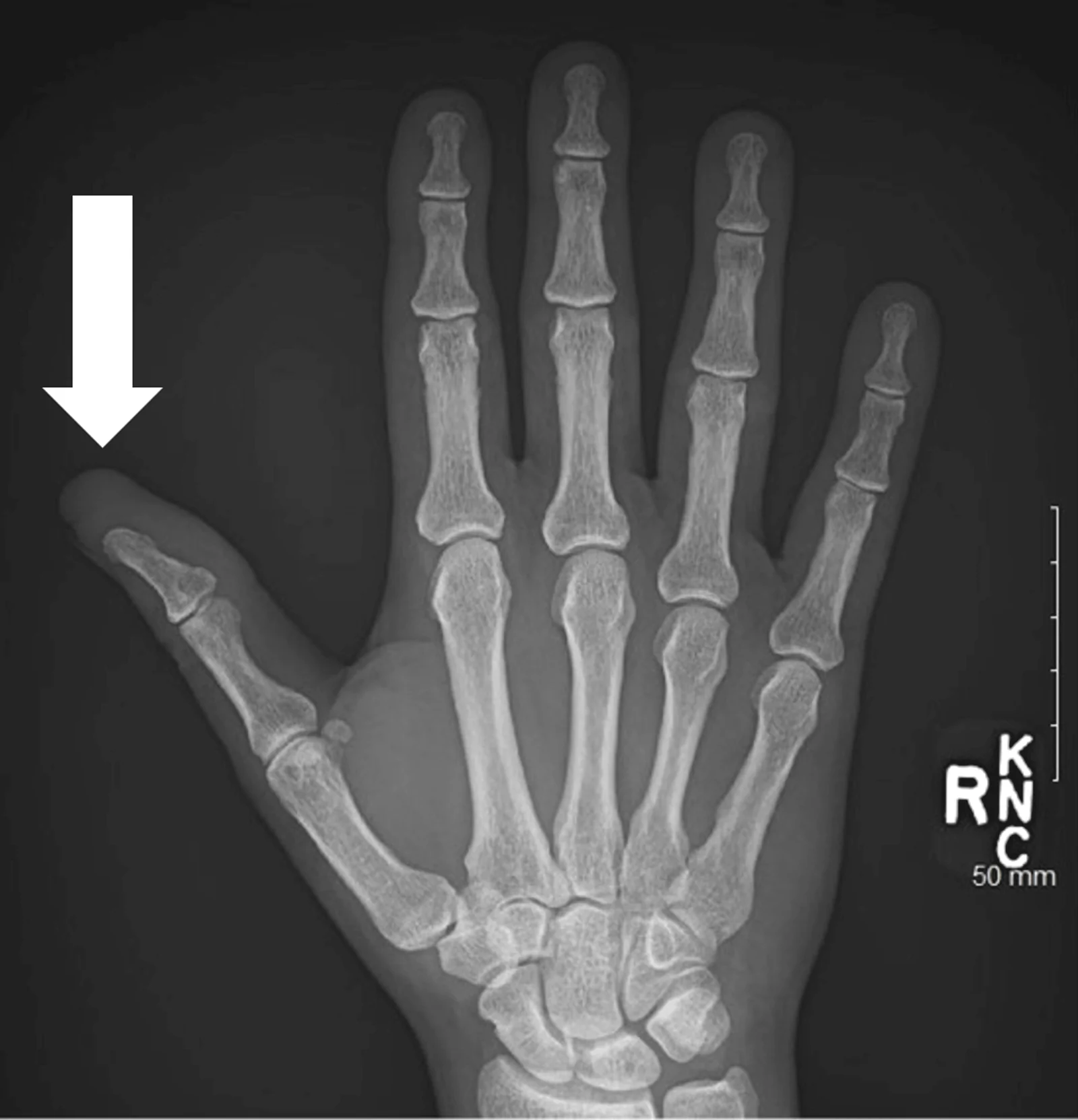
Anteroposterior radiograph of the right hand demonstrating a soft tissue mass at the volar thumb pulp.

**Figure 3:**
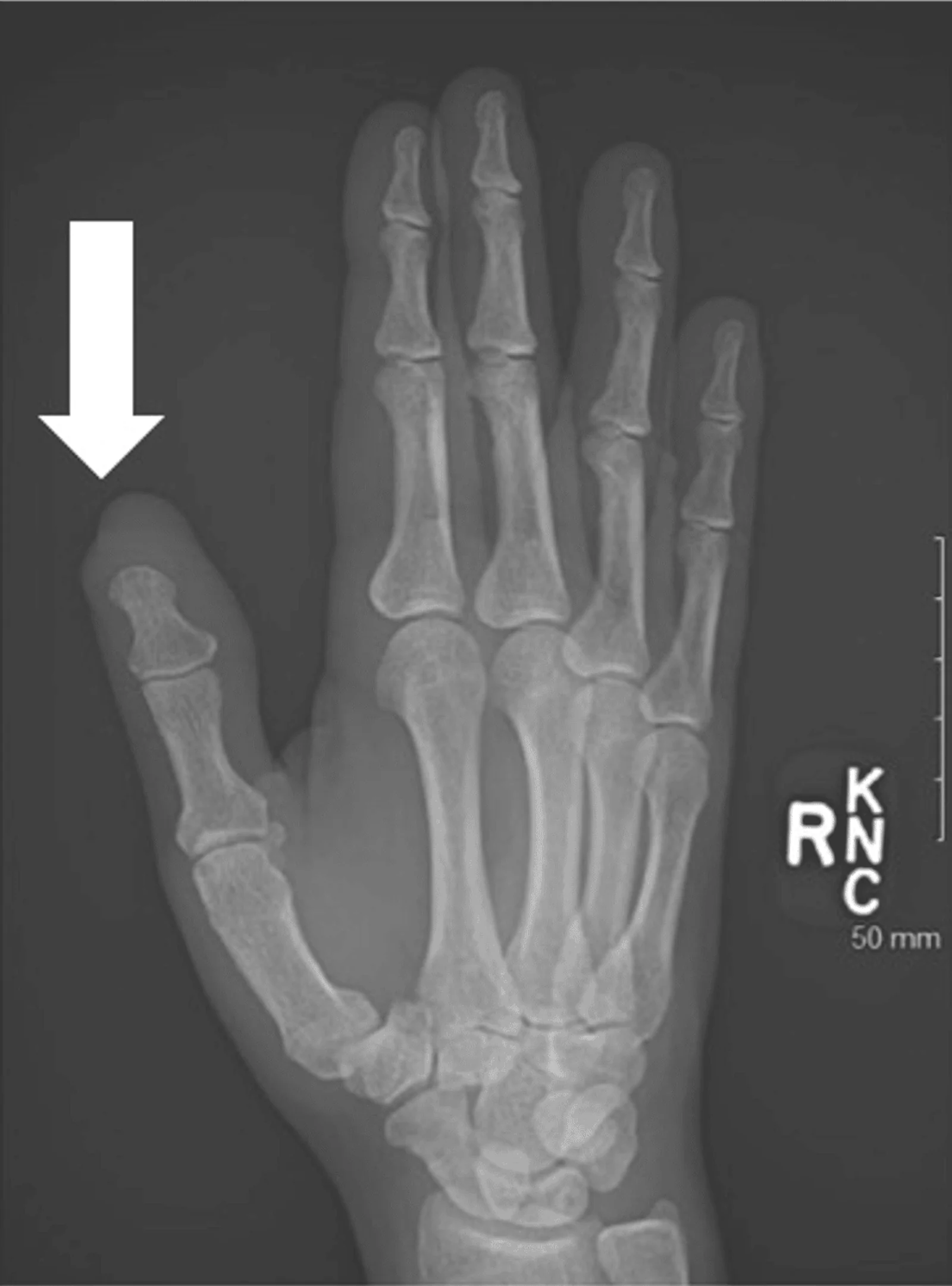
Oblique plain radiograph further delineating the thumb pulp lesion without bony involvement.

**Figure 4:**
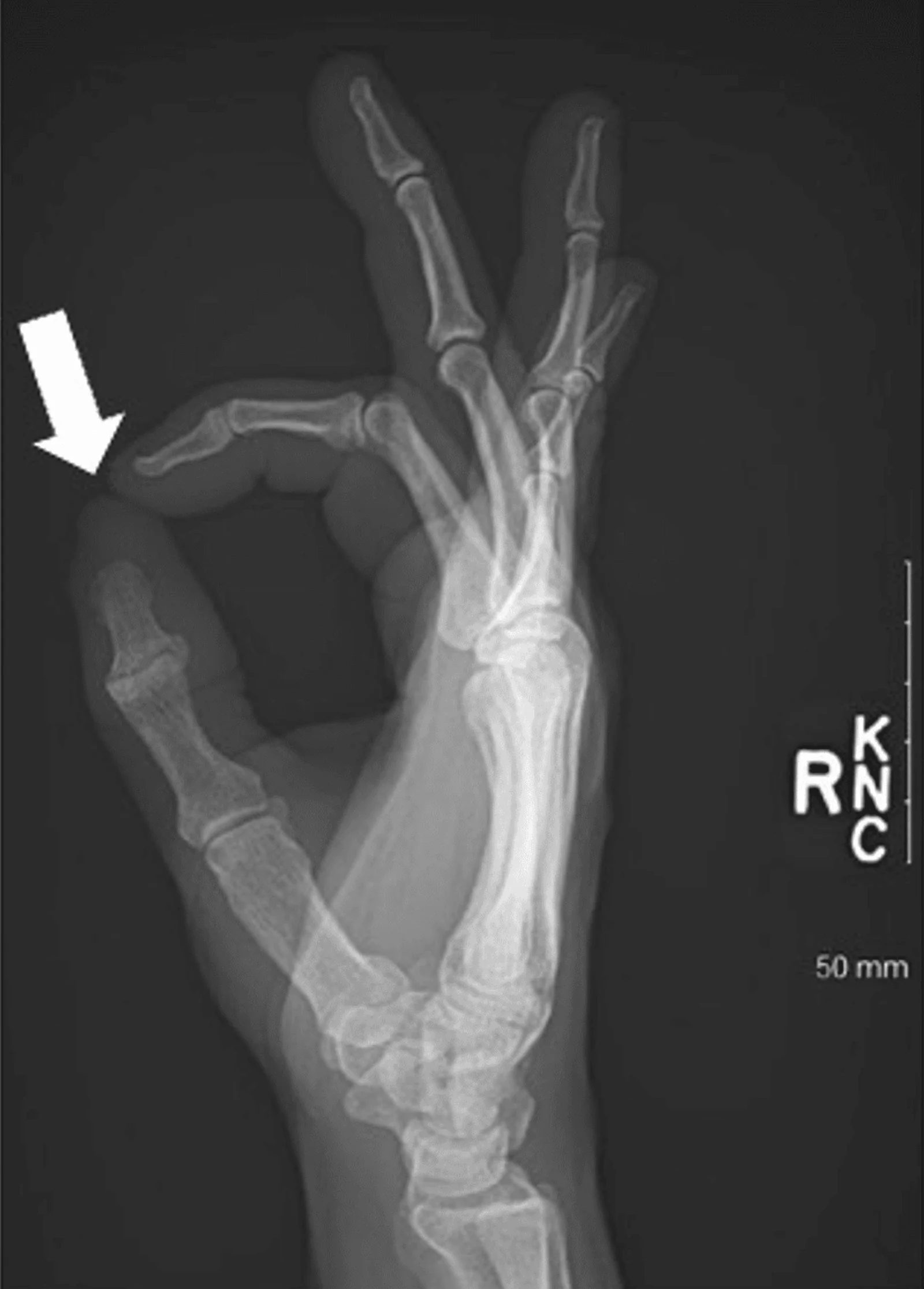
Lateral plain radiograph confirming absence of osseous erosion or periosteal reaction.

Plain radiographs of the right thumb, including anteroposterior, oblique, and lateral views, demonstrated a soft-tissue mass without evidence of osseous involvement or cortical destruction (Figure 2, Figure 3, Figure 4).

**Figure 5:**
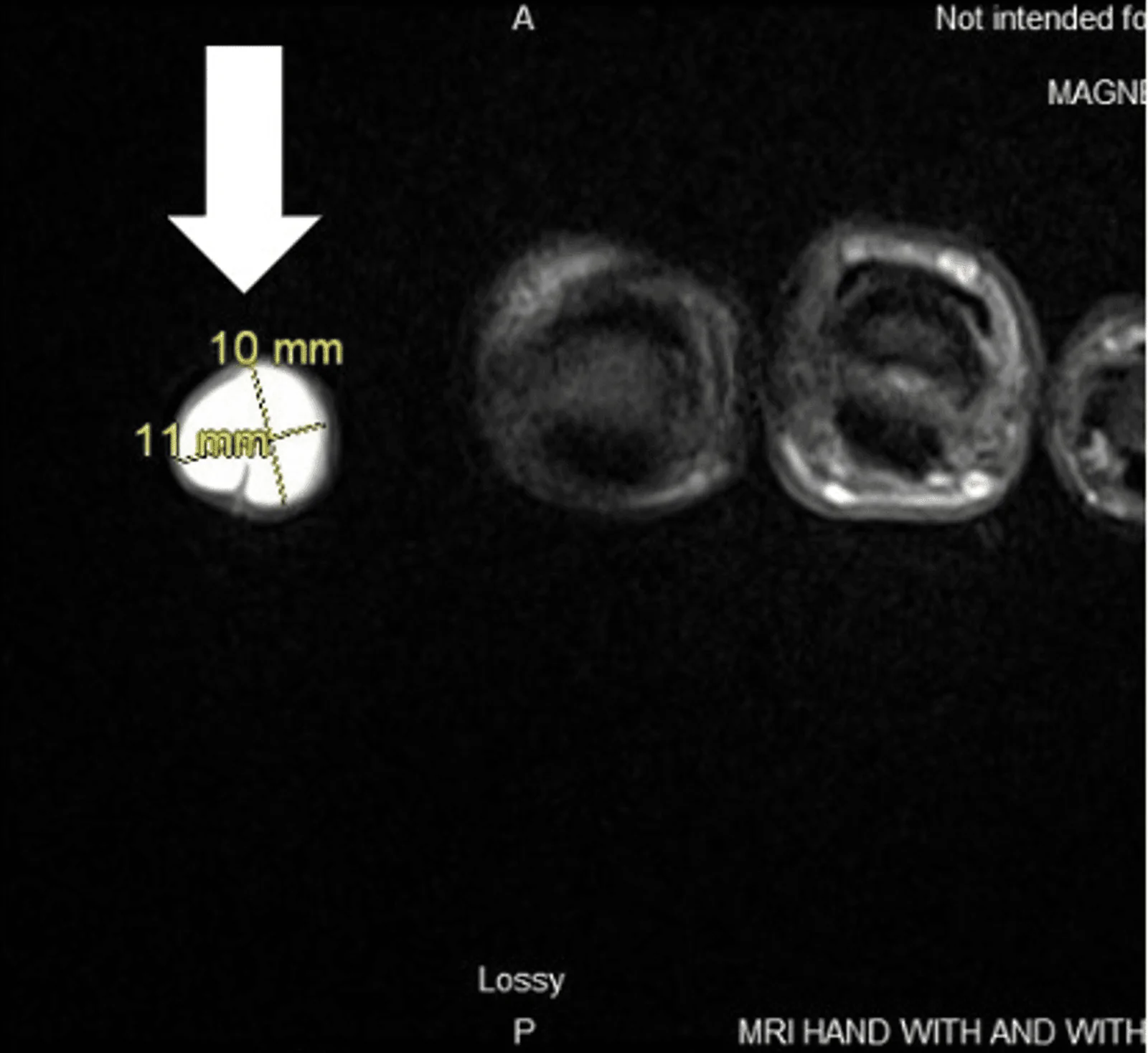
Coronal PD FS magnetic resonance image showing homogeneous high signal intensity of the lesion, leading to a preliminary radiologic impression of epidermal inclusion cyst. PD FS: proton-density fat-saturated

**Figure 6:**
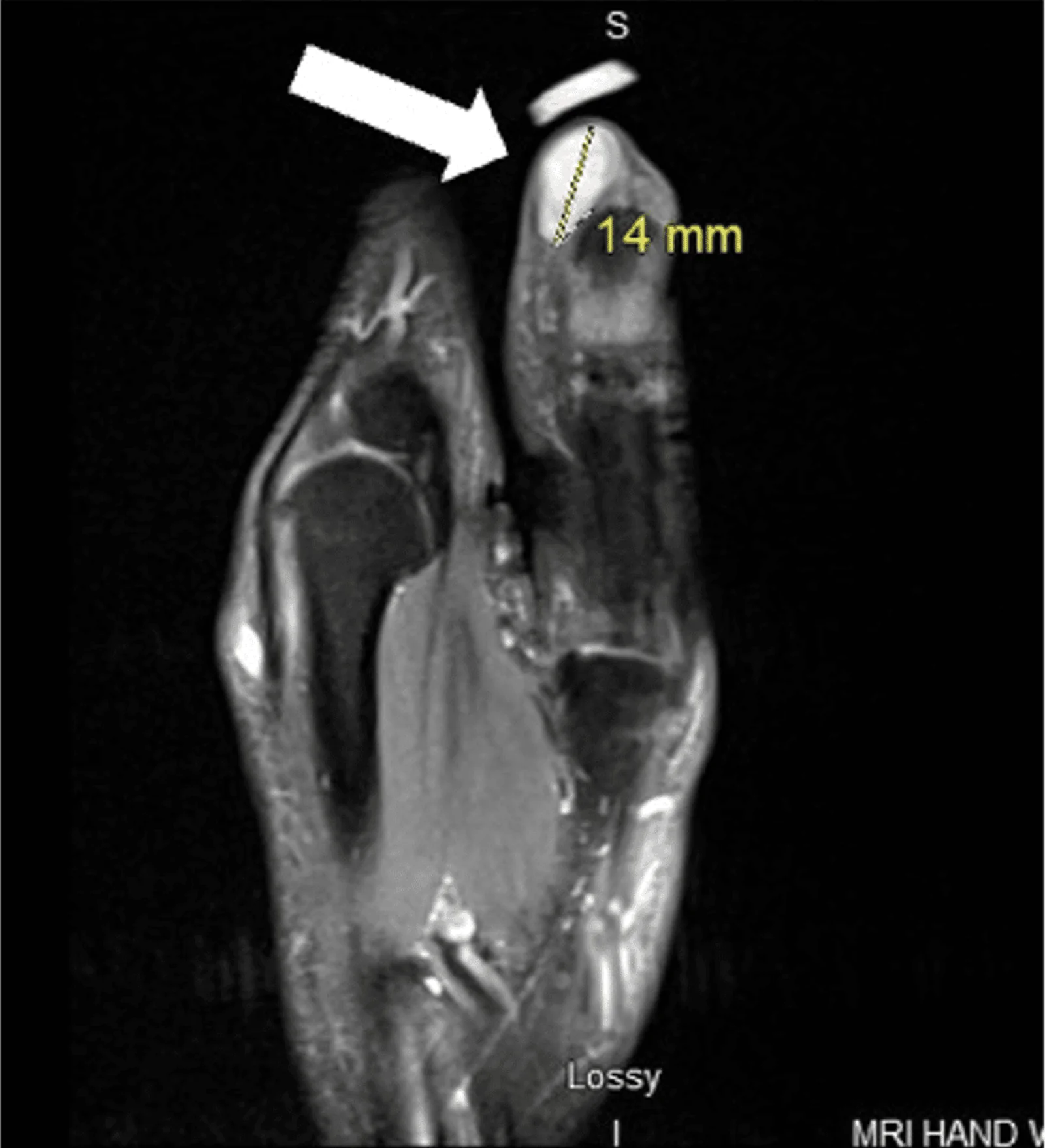
Axial PD FS magnetic resonance image of the right thumb demonstrating a well-circumscribed, homogeneous, high-signal-intensity lesion within the thumb pulp, contributing to its cystic appearance. PD FS: proton-density fat-saturated

**Figure 7:**
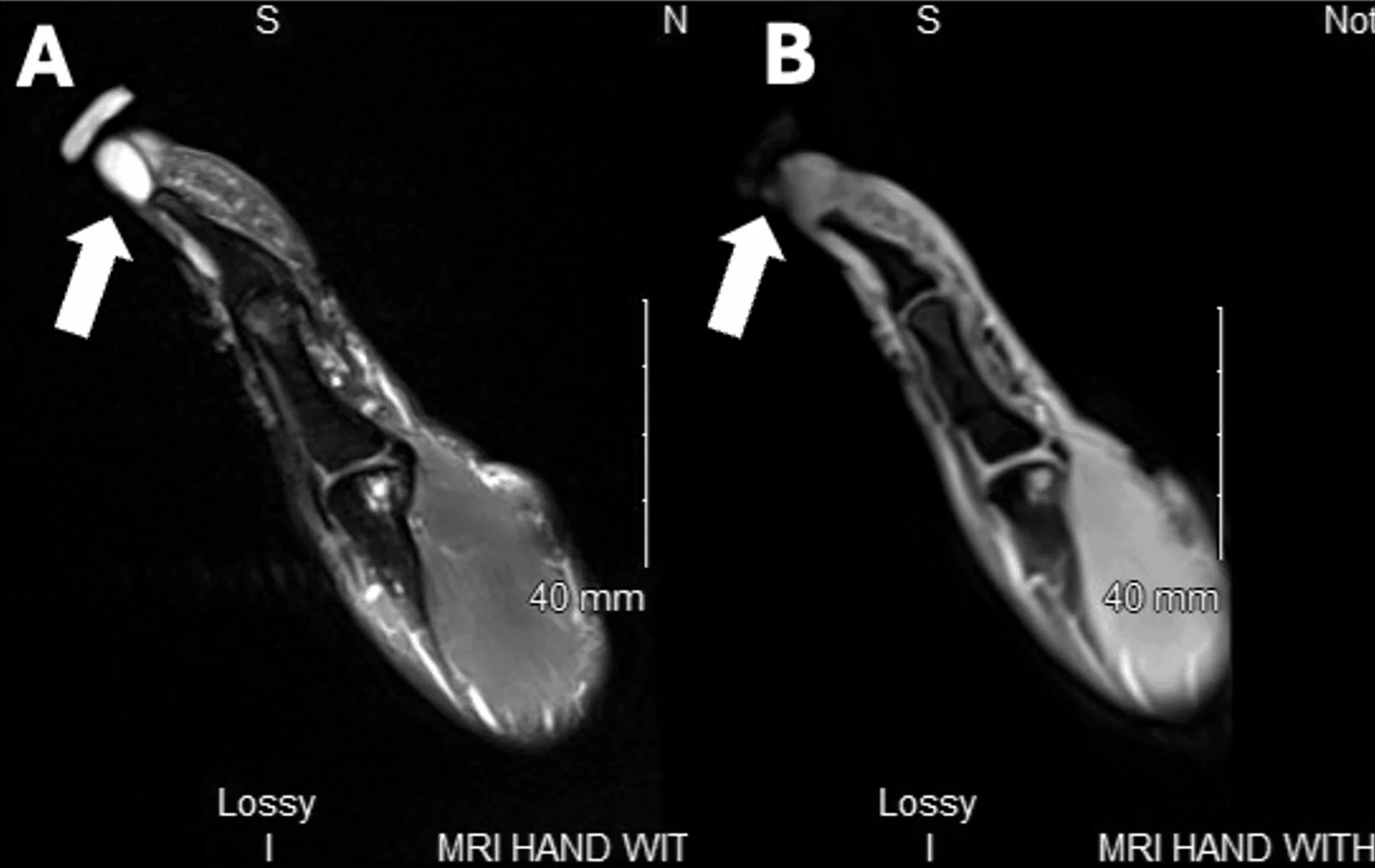
Sagittal precontrast (A) and postcontrast (B) T1-weighted VIBE magnetic resonance images of the right thumb demonstrating the lesion within the thumb pulp without significant postcontrast enhancement. VIBE: volumetric interpolated breath-hold examination

Magnetic resonance imaging with and without contrast was performed at 1.5 T with a 2.0-3.0-mm slice thickness and 10 mL gadolinium-based contrast. Sequences included axial T1 and proton-density fat-saturated (PD FS), coronal T1, T2, and PD FS, sagittal T2 FS, and pre- and postcontrast volumetric interpolated breath-hold examination (VIBE) sequences with subtraction imaging. MRI demonstrated a 1.0 x 1.1 x 1.4 cm T1-hypointense, T2-hyperintense cystic subcutaneous lesion at the tip of the thumb without significant adjacent soft-tissue edema or osseous involvement (Figure 5, Figure 6). Pre- and postcontrast images demonstrated no significant postcontrast enhancement (Figure 7). The flexor and extensor tendons were unremarkable. The radiologist interpreted the findings as most consistent with an EIC or sebaceous cyst.

Intraoperatively, the mass was larger than expected (greater than 1.5 cm) and extended down to the periosteum at the tuft of the distal phalanx. Histopathologic examination subsequently demonstrated a 1.7 x 1.0 x 1.0 cm specimen. The differences among the clinical, radiographic, intraoperative, and pathologist measurements likely reflect the distinct methods used for assessment, as well as the deeper extent of the lesion that was not fully appreciated on physical examination. It was thinly encapsulated and well-circumscribed, allowing for complete removal. The procedure was without complication. No neural involvement was documented. At the two-week and one-month follow-up visits, there was no evidence of tingling, numbness, weakness, or paresthesia. At the six-month follow-up visit, there was no evidence of recurrence.

**Figure 8:**
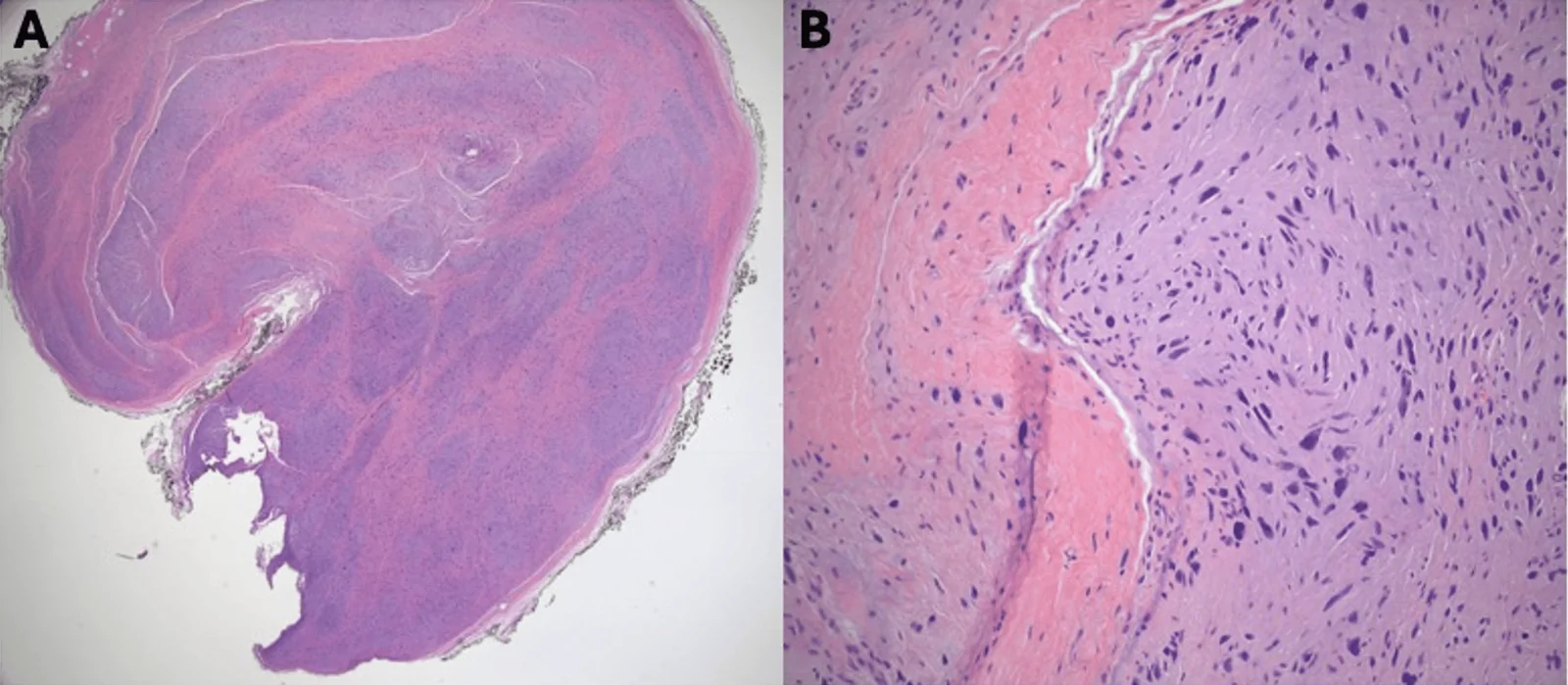
Histopathologic images (A) Low-power view of the mass showing alternating areas of compact (Antoni A) and loosely arranged (Antoni B) foci. The mass is well circumscribed, with no infiltration into surrounding tissue. Hematoxylin and eosin, 20x magnification. (B) High-power view demonstrating persistent compact and loosely arranged foci, with nuclear atypia including anisonucleosis and nuclear membrane irregularities, consistent with degenerative (ancient) change. Hematoxylin and eosin, 200x magnification.

**Figure 9:**
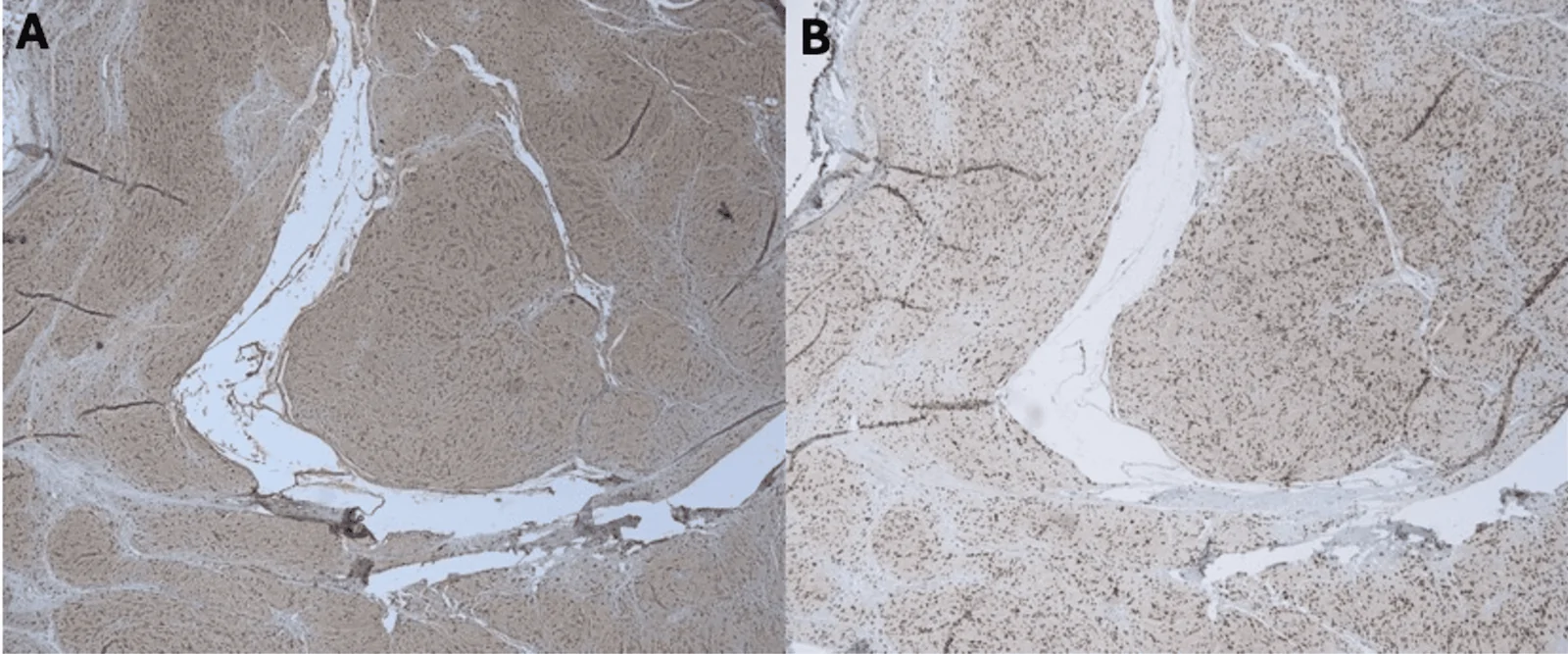
Immunohistochemical staining images. (A) Immunohistochemical staining demonstrating diffuse and strong cytoplasmic and nuclear staining for S100. S100 immunohistochemical stain, 40x magnification. (B) Immunohistochemical staining showing diffuse and strong nuclear staining for SOX10. SOX10 immunohistochemical staining, 40x magnification.

Histopathologic evaluation demonstrated a well-circumscribed, non-infiltrative spindle-cell neoplasm with alternating Antoni A and Antoni B areas and nuclear atypia with anisonucleosis and nuclear membrane irregularities (Figure 8), consistent with degenerative (ancient) change. No additional histopathologic findings regarding mitotic activity or tumor necrosis were reported. Immunohistochemical staining demonstrated diffuse and strong positivity for S100 and SOX10 (Figure 9). No additional immunohistochemical stains were reported. In conjunction with the encapsulated architecture and characteristic morphologic features, these findings support the diagnosis of schwannoma and help distinguish it from other spindle-cell neoplasms, including neurofibroma.

## Discussion

Imaging is a frequently used tool in the evaluation of subcutaneous lesions, helping assess size, depth, involvement of adjacent structures, and occasionally refining the differential diagnosis. For peripheral nerve sheath tumors such as schwannomas, MRI is the imaging modality of choice due to its superior soft tissue resolution [10]. Schwannomas typically appear as well-circumscribed, encapsulated masses that are iso- to hypointense on T1-weighted sequences and show hyperintensity on T2-weighted sequences [11]. They often enhance with contrast, and smaller tumors show homogeneous enhancement, while larger lesions may enhance heterogeneously.

Magnetic resonance imaging can also help differentiate schwannomas from other benign soft tissue tumors. For example, neurofibromas may display a “target sign”, characterized by relatively low central T2 signal intensity with higher peripheral signal intensity, while schwannomas may demonstrate the “split-fat sign”, representing preserved or displaced fat surrounding an intramuscular neurogenic tumor [12,13]. However, these signs are not specific and may be seen in other peripheral nerve sheath tumors. Ancient schwannomas, due to their degenerative changes, can present with variable MRI features [5]. Previously reported ancient schwannomas at other anatomical sites have demonstrated heterogeneous MRI appearances, including cystic or hemorrhagic degeneration and variable enhancement patterns [7]. In contrast, the lesion in our case was small and cystic-appearing without significant postcontrast enhancement, contributing to its radiologic interpretation as a benign cyst. As seen in our case, these imaging variations can lead to diagnostic confusion. For a cystic-appearing lesion of the thumb pulp without significant postcontrast enhancement, the imaging differential includes schwannoma, EIC, and other benign soft-tissue lesions such as fibromas and myxomas. Given the overlap in imaging features, histological examination remains definitive for diagnosis.

For peripheral schwannomas, MRI has a reported sensitivity and specificity of 85% and 50%, respectively [14]. Other imaging modalities include ultrasound and plain radiographs. Ultrasound is commonly used first-line for superficial lesions because of its accessibility. Schwannomas may appear as well-defined, hypoechoic masses with posterior acoustic enhancement [15]. However, ultrasound is often unable to differentiate schwannomas from other similar soft tissue masses [16]. Radiographs, although limited in soft tissue detail, are often obtained first to rule out bony involvement before proceeding to MRI, as was done in our case [17]. While contrast-enhanced MRI remains the preferred imaging modality for preoperative assessment, histopathological evaluation remains the gold standard for definitive diagnosis of schwannomas [18]. Complete surgical excision is a therapeutic approach that may be selected based on tumor location, symptoms, and surgical feasibility.

Ancient schwannomas of the digits are exceedingly rare, with only isolated thumb and fingertip cases reported [19]. In this report, imaging included only CT, and MRI findings were not described. In our case, the lesion’s cystic appearance without significant postcontrast enhancement led radiologists to favor a benign cyst. This atypical MRI appearance may reflect the underlying degenerative histologic features of ancient schwannoma. Histopathology demonstrated alternating Antoni A and Antoni B areas with degenerative nuclear atypia. Prior radiologic-pathologic studies have associated heterogeneous and cystic MRI appearances of schwannomas with a greater proportion of Antoni B tissue, providing a potential explanation for the atypical imaging appearance in this case [7, 12]. EICs themselves are uncommon in the thumb because palmoplantar skin lacks pilosebaceous units. When present, they are typically attributed to traumatic implantation of epidermal elements rather than follicular occlusion. For example, a case report described a 53-year-old carpenter who developed multiple epidermal cysts in the volar thumb skin after decades of repetitive trauma, underscoring both their rarity and their association with chronic injury [20].

This case highlights the potential for atypical MRI features to create diagnostic uncertainty and reinforces the role of histopathologic confirmation for definitive diagnosis.

## Conclusions

This report describes a rare case of an ancient schwannoma of the thumb pulp, a location seldom involved by schwannomas and, to our knowledge, not previously described with detailed MRI findings. In this case, the lesion demonstrated an atypical cystic appearance without significant post-contrast enhancement, mimicking an EIC on MRI. Clinicians should maintain awareness of schwannomas in the differential diagnosis of recurrent or atypical digital lesions and interpret MRI findings alongside the clinical history and imaging characteristics, with histopathological examination remaining the definitive diagnostic standard.

## References

[1] Saad EA, Sati H, Boueri M, Nasr L, Fawaz A, Emmanuel N (2024). Cutaneous schwannomas: clinical manifestations, diagnosis and management: a review. Curr Dermatol. Rep.

[2] He S, Shan G (2024). A clinical and histopathological analysis of 45 cases of cutaneous neurilemmoma (Schwannomas). J Biosci Med.

[3] Zelger BG, Steiner H, Kutzner H, Rütten A, Zelger B (1997). Verocay body--prominent cutaneous schwannoma. Am J Dermatopathol.

[4] Venkataramana CG, Gupta S, Nayak R, Km S, Rai S, Rao R (2024). Comprehensive study of ancient schwannoma: Exploring histomorphological diversity and diagnostic challenges. Rare Tumors.

[5] Unnithan AKA, Mohan A, Geetha Gopal K (2022). A case of ancient schwannoma of arm with profuse vascularity [PREPRINT]. Res Square.

[6] Gupta R, Cai Y, Li L, Galan M, Datiashvili RO (2023). Rare case of a cutaneous fingertip schwannoma: a case report and review of literature. Eplasty.

[7] Isobe K, Shimizu T, Akahane T, Kato H (2004). Imaging of ancient schwannoma. AJR Am J Roentgenol.

[8] Argenyi ZB, Balogh K, Abraham AA (1993). Degenerative ("ancient") changes in benign cutaneous schwannoma. A light microscopic, histochemical and immunohistochemical study. J Cutan Pathol.

[9] Plotkin SR, Messiaen L, Legius E (2022). Updated diagnostic criteria and nomenclature for neurofibromatosis type 2 and schwannomatosis: an international consensus recommendation. Genet Med.

[10] Ko JY, Kim JE, Kim YH, Ro YS (2009). Cutaneous plexiform schwannomas in a patient with neurofibromatosis type 2. Ann Dermatol.

[11] Attia EA, Yassin M, Lasheen MA, Salem SA, Khafagy NH (2011). Multiple isolated cutaneous plexiform schwannomas. Indian J Dermatol Venereol Leprol.

[12] Crist J, Hodge JR, Frick M, Leung FP, Hsu E, Gi MT, Venkatesh SK (2017). Magnetic resonance imaging appearance of schwannomas from head to toe: a pictorial review. J Clin Imaging Sci.

[13] Zhang Z, Deng L, Ding L, Meng Q (2015). MR imaging differentiation of malignant soft tissue tumors from peripheral schwannomas with large size and heterogeneous signal intensity. Eur J Radiol.

[14] Istefan E, Belstock J, Dahlin LB, Nyman E (2023). Surgery of Schwannoma in the upper limb - sensitivity and specificity of preoperative magnetic resonance imaging and relation between tumour size and symptoms. BMC Musculoskelet Disord.

[15] Tsai WC, Chiou HJ, Chou YH, Wang HK, Chiou SY, Chang CY (2008). Differentiation between schwannomas and neurofibromas in the extremities and superficial body: the role of high-resolution and color Doppler ultrasonography. J Ultrasound Med.

[16] Lee SJ, Yoon ST (2017). Ultrasonographic and clinical characteristics of schwannoma of the hand. Clin Orthop Surg.

[17] Albert P, Patel J, Badawy K, Weissinger W, Brenner M, Bourhill I, Parnell J (2017). Peripheral nerve schwannoma: a review of varying clinical presentations and imaging findings. J Foot Ankle Surg.

[18] Karna MB, Kinanta PB, Aprilya D (2023). Recurrent schwannoma of digital nerve on both hands: a very rare case report. Int J Surg Case Rep.

[19] Matsumine H, Takeuchi M (2015). Ancient schwannoma of the thumb pulp. Plast Reconstr Surg Glob Open.

[20] Kim HJ, Koh SH, Jung SW, Yang J, Lim H (2016). Multiple epidermal cysts in the volar skin of the thumb. Arch Plast Surg.

